# Decreased Expression of Transcription Elongation Factor A-Like *7* Is Associated with Gastric Adenocarcinoma Prognosis

**DOI:** 10.1371/journal.pone.0054671

**Published:** 2013-01-25

**Authors:** Chun-yu Huang, Yong-ming Chen, Jing-jing Zhao, Yi-bing Chen, Shan-shan Jiang, Shu-mei Yan, Bai-wei Zhao, Ke Pan, Dan-dan Wang, Lin Lv, Yuan-fang Li, Wei Wang, Zhi-wei Zhou, Jian-chuan Xia

**Affiliations:** 1 Department of Gastric and Pancreatic Surgery, Sun Yat-sen University Cancer Center, Guangzhou, People's Republic of China; 2 State Key Laboratory of Oncology in South China and Department of Experimental Research, Sun Yat-sen University Cancer Center, Guangzhou, People's Republic of China; 3 Department of Pathology, Sun Yat-sen University Cancer Center, Guangzhou, People's Republic of China; National Cancer Center, Japan

## Abstract

**Background:**

We sought to investigate the expression levels and prognosis value of TCEAL7 in primary gastric cancer.

**Methods and Results:**

We investigated TCEAL7 and other homologous five members of the TCEAL family expression in normal gastricepithelial cell line and gastric cancer cell lines using real-time quantitative PCR. Furthermore, we examined the expression of TCEAL7 in 39 paired cancerous and matched adjacent noncancerous gastric mucosa tissues by real-time quantitative PCR and western blotting. Moreover, we analyzed TCEAL7 expression in 406 gastric cancer patients using immunohistochemistry. The relationships between the TCEAL7 expression levels, the clinicopathological factors, and patient survival were investigated. RT- qPCR data showed that mRNA expression level of TCEAL7 was significantly lower in the gastric cancer cell lines comparing with the levels of other five members of the TCEAL family. Results also revealed decreased TCEAL7 mRNA (P = 0.025) and protein (P = 0.012) expression in tumor tissue samples compared with matched adjacent non-tumor tissue samples. Immunohistochemical staining data showed that TCEAL7 expression was significantly decreased in 43.3% of gastric adenocarcinoma cases. The result also showed that the low TCEAL7 expression was significantly correlated with female, larger tumor size, higher histological grade and worse nodal status. Kaplan–Meier survival curves revealed that the reduced expression of TCEAL7 was associated with a poor prognosis in gastric adenocarcinoma patients (P<0.001). Based on a univariate analysis that included all 406 patients, TCEAL7 expression was found to have statistically significant associations with overall survival (P<0.001). Multivariate analysis also demonstrated that TCEAL7 expression (P = 0.009), age, tumor size, histological grade, lymphovascular invasion, T stage, N stage and M stage were independent risk factors in the prognosis of gastric cancer patients.

**Conclusions:**

Our study suggests that TCEAL7 might serve as a candidate tumor suppressor and a potential prognostic biomarker in gastric carcinogenesis.

## Introduction

Although the incidence of gastric cancer has decreased over the past few decades, it remains the fourth most common cancer in the world and the second most common cause of cancer-related deaths [1]. In China, gastric cancer was predicted to rank as the third most common cancer in 2005, with 0.3 million deaths and 0.4 million new cases reported [2]. Treatment of gastric cancer includes a combination of surgery, chemotherapy, and radiation therapy. Nevertheless, nearly 60% of the patients affected succumb to gastric cancer after a curative resection alone or after adjuvant therapy [3]. Multi-modal and individualized treatments are often based on the TNM staging status. However, the treatment response and prognosis vary in gastric cancer patients of the same stage using the same therapeutic strategy. Therefore, finding a suitable marker to predicate the prognosis of gastric cancer is necessary.

Several candidate genes have emerged based on the accumulating evidence of differential expression or epigenetic silencing in tumors. One down-regulated gene is transcription elongation factor A (SII)-like 7 (TCEAL7), which is located on the X chromosome and encodes a cell death regulatory protein that is inactivated by methylation [4], [5]. Down-regulation of TCEAL7 has been associated with increased NF-κB activity, higher levels of expression of the pro-proliferative genes cyclin D1 and c-Myc as well as the pro-angiogenic genes IL-6, IL-8, and VEGF [6]. TCEAL7 shares amino acid sequence homology with several other pro-apoptotic proteins [7], and its expression is lost in over 90% of primary ovarian tumors and 100% of the cell lines tested compared to adjacent genes on the X chromosome [4]. TCEAL7 is also down-regulated in breast, brain, and prostate cancer, suggesting that it plays an important role in carcinogenesis possibly through uncontrolled expression of cyclin D1 and c-Myc [6]. However, to the best of our knowledge, no previous reports exist concerning the expression status of TCEAL7 in primary gastric cancer, and the prognostic value of this protein in gastric cancer has not yet been assessed.

In this study, we aimed to analyze the TCEAL7 expression level in gastric cancer using real-time quantitative RT-PCR (reverse transcription polymerase chain reaction), western blotting and immunohistochemistry. Furthermore, we identified the relationship between TCEAL7 expression and the clinicopathological features of the disease and evaluated its prognostic value for post-resection survival in gastric cancer.

## Results

### RT-qPCR analysis of TCEAL1, TCEAL3, TCEAL4, TCEAL5, TCEAL7 and TCEAL8 expression in normal gastricepithelial cell line and gastric cancer cell lines

**Figure 1:**
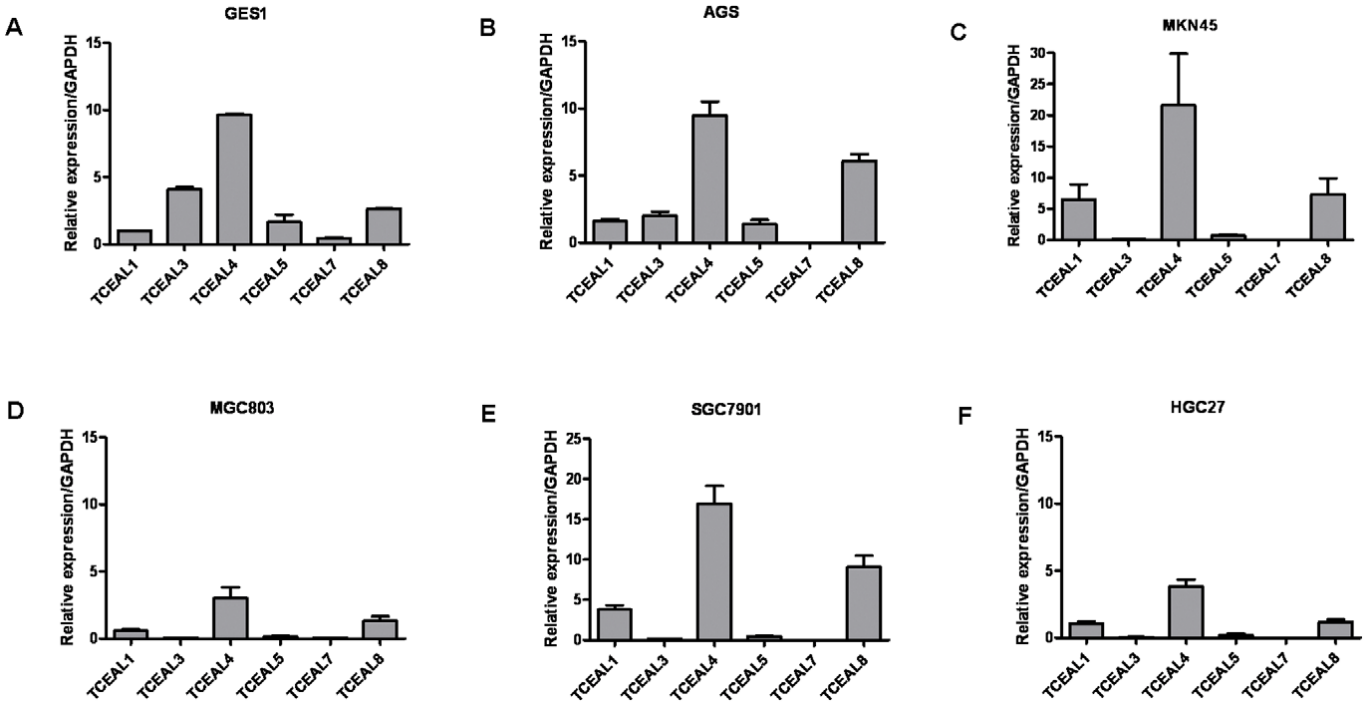
RT-qPCR analysis of TCEAL1, TCEAL3, TCEAL4, TCEAL5, TCEAL7 and TCEAL8 expression in normal gastricepithelial cell line and gastric cancer cell lines. Relative expression of TCEAL7 in normal gastricepithelial cell line GES1 (**A**) and gastric cancer cell lines including AGS (**B**), MKN45 (**C**), MGC803 (**D**), SGC7901 (**E**) and HGC27 (**F**). The TCEAL7 expression level was significantly lower in the gastric cancer cell lines comparing with the levels of TCEAL1, TCEAL3, TCEAL4, TCEAL5 and TCEAL8. Furthermore, the TCEAL7 mRNA expression was down-regulated in the AGS, MKN45, MGC803, SGC7901 and HGC27 cells compared with the normal gastricepithelial cell line GES1.

A real-time quantitative PCR was performed on normal gastricepithelial cell line GES1 and gastric cancer cell lines including AGS, MKN45, MGC803, SGC7901 and HGC27 to determine the mRNA levels of TCEAL1, TCEAL3, TCEAL4, TCEAL5, TCEAL7 and TCEAL8. The TCEAL7 expression level was significantly lower in the gastric cancer cell lines comparing with the levels of TCEAL1, TCEAL3, TCEAL4, TCEAL5 and TCEAL8 (Figure 1). Furthermore, TCEAL7 expression level was significantly lower in the gastric cancer cell lines than in the GES1normal gastricepithelial cell line (Figure 1). Additionally, the MKN45, MGC803, SGC7901 and HGC27 gastric cell lines showed decreased TCEAL3 transcript levels relative to the GES1 and AGS cell lines (Figure 1).

### TCEAL7 mRNA expression analyzed with qRT-PCR

**Figure 2:**
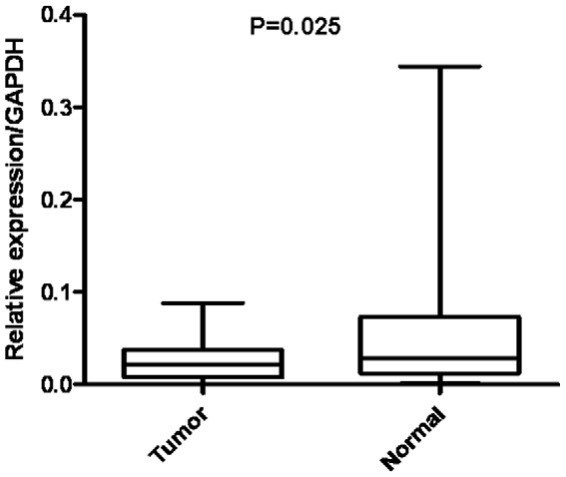
RT-qPCR analysis of TCEAL7 expression in gastric cancer patients. Relative expression of TCEAL7 in gastric cancer tumor tissues compared to adjacent non-tumor tissues (n = 39) assessed by qRT-PCR (P = 0.025).

The transcriptional levels of TCEAL7 were determined with qRT-PCR assays using 39 pairs of resected specimens (tumor tissue samples and matched adjacent non-tumor tissue samples) from gastric cancer patients. The TCEAL7 mRNA levels were significantly reduced in 27 (69.2%) tumor tissue samples, compared with its levels in the adjacent non-tumor tissue samples (P = 0.025, Figure 2), whereas there were only 5 (12.8%) noncancerous tissue samples' TCEAL7 expression were lower than matched tumor tissue samples.

### TCEAL7 expression analyzed by Western blotting

**Figure 3:**
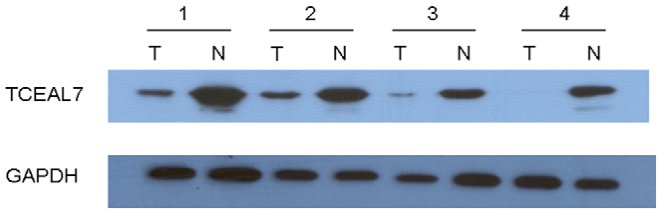
Western blotting analysis of TCEAL7 expression in gastric cancer patients. Relative expression of TCEAL7 in gastric cancer tumor (T) tissues compared to adjacent non-tumor (N) tissues (n = 39) assessed by western blotting, (P = 0.012).

The TCEAL7 protein levels in the resected gastric cancer samples were determined by Western blotting. The results showed a TCEAL7 band at 22 kDa. Consistent with the quantitative real-time PCR results, a decrease in TCEAL7 expression was observed in 28 (71.8%) of the gastric tumor tissues compared with matched adjacent non-tumor tissues (P = 0.012, Figure 3), whereas there were only 6 (15.4%) noncancerous tissue samples' TCEAL7 expression were lower than matched tumor tissue samples.

### Immunohistochemical analysis of TCEAL7 expression in gastric cancer tissue samples and its relationship with the clinicopathological features

**Figure 4:**
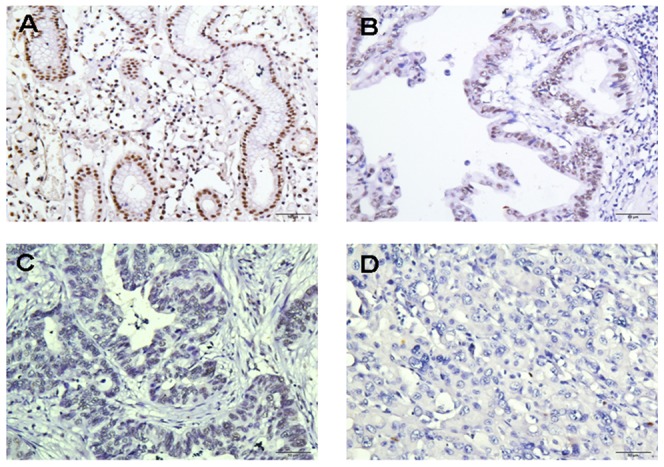
Immunohistochemical detection of the TCEAL7 protein expression in gastric cancer and surrounding non-tumor tissues. (A) Normal gastric tissues, scored as TCEAL7 (+++); (B) Well-differentiated gastric cancer, scored as TCEAL7 (++);(C) Moderately differentiated gastric cancer, scored as TCEAL7 (+); (D) poorly differentiated gastric cancer, scored as TCEAL7 (−). Original magnification: A–D×200.

In order to confirm the molecular biological findings and investigate the clinicopathological the prognostic roles of TCEAL7 expression, we performed immunohistochemical analysis in 406 paraffin-embedded gastric cancer sections. The positive expression of TCEAL7 was localized to the nucleus. Among the 406 gastric cancer samples, 230 (56.7%) showed high TCEAL7 expression (TCEAL7 ++ or TCEAL7 +++), whereas the remaining 176 cases (43.3%) displayed low TCEAL7 expression (TCEAL7− or TCEAL7 +) (Figure 4, Table 1). Normal gastric tissues showed the strongest TCEAL7 positive staining (Figure 4A). Well-differentiated cases showed strongly positive TCEAL7 expression (TCEAL7 ++) (Figure 4B), moderately-differentiated cases showed weakly positive expression (TCEAL7 +) (Figure 4C), and the most poorly differentiated cases often showed no detectable TCEAL7 expression (TCEAL7−) (Figure 4D).

**Table 1:** Relationship between TCEAL7 expression (localized to the nucleus) and clinicopathologic features of patients with gastric cancer.

Variables	Number (%)	TCEAL7 expression		*P* value
		Low (%)	High (%)	
**Age(years)**				0.119
<60	230(56.7)	92(40.0)	138(60.0)	
≥60	176(43.3)	84(47.7)	92(52.3)	
**Gender**				0.001*
Male	276(68.0)	104(37.7)	172(62.3)	
Female	130(32.0)	72(55.4)	58(44.6)	
**Tumor size (cm)**				0.041*
≤2.5	51(12.6)	14(27.5)	37(72.5)	
2.5–5.0	210(51.7)	93(44.3)	117(55.7)	
>5.0	145(35.7)	69(47.6)	76(52.4)	
**histological grade**				0.017*
G1/G2	156(38.4)	56(35.9)	100(64.1)	
G3	250(61.6)	120(48.0)	130(52.0)	
**Tumor location**				0.052
Proximal	236(58.1)	103(43.6)	133(56.4)	
Distant	153(37.7)	61(39.9)	92(60.1)	
Total	17(4.2)	12(70.6)	5(29.4)	
**Lymphovascular invasion**				0.293
Yes	39(9.6)	20(51.3)	19(48.7)	
No	367(90.4)	156(42.5)	211(57.5)	
**Tumor invasion (T)**				0.189
T1/T2/T3	146(36.0)	57(39.0)	89(61.0)	
T4a/T4b	260(64.0)	119(45.8)	141(54.2)	
**Nodal status (N)**				0.029*
N0	115(28.3)	40(34.8)	75(65.2)	
N+	291(71.7)	136(46.7)	155(53.3)	
**Metastasis status (M)**				0.891
M0	354(87.2)	153(43.2)	201(56.8)	
M1	52(12.8)	23(44.2)	29(55.8)	

* Statistically significant (*P*<0.05).

Based on the categories that we defined in the afore mentioned methods, the data showed that the low TCEAL7 expression was significantly correlated with female (r = 0.171, P = 0.001), larger tumor size (r = 0.123, P = 0.041), higher histological grade (r = 0.091, P = 0.017) and worse nodal status (r = 0.102, P = 0.029)

### Correlation between TCEAL7 expression based on immunohistochemistry and patient survival

**Figure 5:**
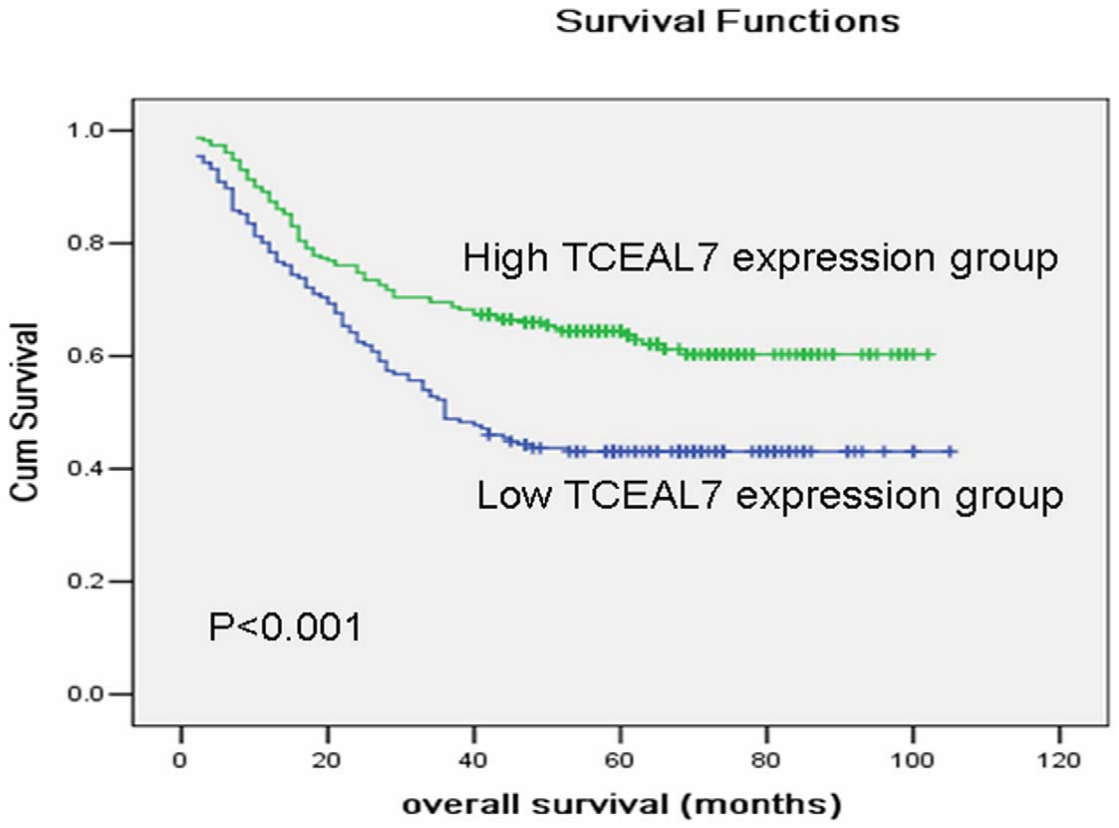
Kaplan–Meier survival curves of gastric cancer patients (n = 406) after surgical resection. Decreased TCEAL7 expression correlated with poor patient survival. Patients in high TCEAL7 expression group exhibited significantly better survival than the low TCEAL7 expression group (log-rank test: *P*<0.001).

The median survival time of the 406 gastric cancer patients was 46 months (range 2–105 months). The overall survival rate and the 5-year survival rate in the high TCEAL7 expression group were significantly improved compared to the low expression group [64.4% vs. 43.1% (5-year survival rate), P<0.001, Figure 5].

### Univariate and multivariate analyses

Univariate and multivariate analyses were performed to compare the impact of TCEAL7 expression and other clinicopathological parameters on prognosis. Based on a univariate analysis that included all 406 patients, 8 factors were found to have statistically significant associations with overall survival; these factors included age, tumor size, histological grade, lymphovascular invasion, TCEAL7 expression, and the T, N and M stage based on the 7th edition of the UICC TNM classification (Table 2). All 8 factors were included in a multivariate Cox proportional hazards model to adjust for the effects of the covariates. Based on this model, age, tumor size, histological grade, lymphovascular invasion, TCEAL7 expression, T stage, N stage and M stage remained independent prognostic factors (Table 2).

**Table 2:** Univariate and multivariate survival analysis of clinic-pathologic variables in 406 cases of gastric carcinoma patients.

Variables	*n* a	Univariate analyses			Multivariate analyses		
		HR	(95% CI)	*P* value	HR	(95% CI)	*P* value
**Age (years)**				0.033*			0.028*
<60	230	1.000			1.000		
≥60	176	1.367	1.025–1.882		1.396	1.037–1.879	
**Gender**				0.376			
Male	276	1.000					
Female	130	1.148	0.847–1.556				
**Tumor size**				<0.001*			<0.001*
<2.5 cm	51	1.000			1.000		
2.5–5 cm	210	4.002	1.748–9.163		2.441	1.052–5.663	
>5 cm	145	6.483	2.828–14.866		3.767	1.617–8.775	
**histological grade**				0.012*			0.007*
G1/G2	156	1.000			1.000		
G3	250	1.488	1.093–2.027		1.553	1.125–2.143	
**Tumor location**				0.084			
Proximal	236	1.000					
Distant	153	0.538	0.382–0.757				
Total	17	1.806	0.972–3.354				
**Lymphovascular invasion**				<0.001*			<0.001*
No	367	1.000			1.000		
Yes	39	2.956	1.995–4.379		2.267	1.511–3.400	
**Tumor invasion**				<0.001*			0.004*
T1/T2/T3	146	1.000			1.000		
T4a/T4b	260	2.561	1.810–3.624		1.701	1.190–2.432	
**Nodal status**				<0.001*			0.001*
N0	115	1.000			1.000		
N+	291	2.838	1.897–4.245		1.993	1.323–3.002	
**Metastasis status**				<0.001*			<0.001*
M0	354	1.000			1.000		
M1	52	5.151	3.677–7.215		3.527	2.492–4.991	
**TCEAL7**				<0.001*			0.009*
High	230	1.000			1.000		
Low	176	1.950	1.227–2.760		1.658	1.116–2.108	

HR, hazard ratio; CI, confidence interval;

a Numbers of cases in each group;

* Statistically significant (*P*<0.05).

## Discussion

In this study, we investigated the expression of a proapoptotic nuclear protein, TCEAL7 in gastric cancer. We found that the expression of TCEAL7 was significantly reduced at both mRNA and protein levels in gastric cancer compared with normal gastric mucosa. Furthermore, low expression of TCEAL7 was associated with larger tumor size, higher histological grade and N stage. More interesting, we also found that low expression of TCEAL7 was more common in female. Concerning the expression status of TCEAL7 associated with gender has not reported so far. However, many studies had indicated a functional significance of the downregulation of TCEAL7 in oncogenesis of various female cancers [4], [6], [8], [9]. We speculate that the low expression of TCEAL7 might be associated with female hormones, but the mechanism warrants further investigation. We also found that gastric cancer patients with low expression of TCEAL7 showed shorter postsurgical survival than high TCEAL7 expression patients.

TCEAL7 is a newly-identified proapoptotic nuclear protein that shares amino-acid sequence homology with the TCEAL1 (p21/SIIR/pp21) and the pp21 homolog (WBP5/TCEAL6) [4]. Experimental data demonstrated that TCEAL7 protein represses cellular transformation by negatively modulating Myc and NF-κB [6], [8], [10], whereas low expression of TCEAL7 promoted the survival of malignant transformed cells, leading to the development of cancer [4], [9], [11], [12]. It was also found that TCEAL7 expression was lost in a range of cancer cells, including ovarian, cervical, breast, brain and lung cancer cell lines [6]. These findings, collecting with our data that expression of TCEAL7 was low in most gastric cancer samples, suggesting the potential tumor suppressor role of TCEAL7 as previously reported [4], [6], [8]. However, the detailed mechanisms of TCEAL7 in the carcinogenesis of stomach remain to be further elucidated in the future.

Up to date, there are few studies about the expression of TCEAL7 in cancer in large sample size. Chien J et al. have found that expression of TCEAL7 is low in more than 90% primary ovarian cancer samples and cell lines [4]. The low expression of TCEAL7 is mainly due to promoter hypermethylation. Like our findings, expression analysis through the data from the Oncomine database also indicated that the expression of TCEAL7 was downregulated in cancers and its downregulation was associated with higher grade, metastatic or more aggressive phenotype in a range of different tissue-originated cancers [6]. These data suggested that the low expression of TCEAL7 might facilitate the invasion and metastasis of cancer. Nevertheless, the molecular biological functions of TCEAL7 in the progression, invasion and local metastasis of gastric cancer need to be further investigated.

Aside from TCEAL7, there are other homologous five members of the TCEAL family, *i.e.* TCEAL1, TCEAL3, TCEAL4, TCEAL5 and TCEAL8, the expression and functions of which are rarely studied. It is reported that TCEAL1 was involved in the apoptosis of human cancer cells [13]. Microarray study showed that the expression of TCEAL1 was markedly decreased in esophageal squamous cell carcinoma samples compared to matched normal samples [14]. Akaishi J et al. have found that the expression of TCEAL4 was downregulated in anaplastic thyroid cancer compared to either normal thyroid tissues or papillary and follicular thyroid cancer tissues [15]. In the present study, we also examined the expression of other TCEAL family members in gastric cancer cell lines. We found that the TCEAL7 expression level was significantly lower in all the cell lines comparing with the levels of TCEAL1, TCEAL3, TCEAL4, TCEAL5 and TCEAL8. Additionally, TCEAL7 expression level was significantly lower in the gastric cancer cell lines than in the GES1normal gastricepithelial cell line, indicating that down-regulated TCEAL7 expression may play a role in gastric cancer development. These interesting findings suggested the different roles of TCEAL family members in different tissue-originated and different histological malignancies.

In conclusion, this is the first report that the low expression of TCEAL7 was associated with gastric adenocarcinoma prognosis. We confirmed that TCEAL7 might serve as a candidate tumor suppressor and prognostic biomarker in gastric carcinogenesis. In future studies, we are planning to examine 406 gastric adenocarcinoma surgical patients to further validate TCEAL7 for survival prediction of gastric adenocarcinoma surgical patients. The mechanism involved in the biological function of TCEAL7 in gastric cancer warrants further investigation. Moreover, we expect that TCEAL7 may function as a useful target for new therapeutic interventions against gastric adenocarcinoma.

## Materials and Methods

### Ethics statement

The research was approved by the Ethics Committee of Sun Yat-sen University Cancer Center and written informed consent was obtained from each patient involved in the study.

### Human tissue sample*s*

A total of 39 paired cancerous and matched adjacent noncancerous gastric mucosa tissues were collected from gastric cancer patients undergoing gastrectomy at Sun Yat-sen University Cancer Center from 2010 to 2011. After surgical resection, fresh tissues were immediately immersed in RNAlater (Ambion, Inc., USA) to avoid RNA degradation, stored at 4°C overnight to allow thorough penetration of RNAlater into the tissue and then frozen at −80°C until the RNA and protein extraction was performed. Another 406 paraffin-embedded primary gastric carcinoma samples that had been collected between 2003 and 2006 were obtained from the Sun Yat-sen University Cancer Center. None of these patients had received radiotherapy or chemotherapy prior to surgery. The histopathological type and stage of the gastric cancer were determined according to the criteria of the World Health Organization classification and the TNM stage set out by the Union for International Cancer Control.

### Cell lines and culture conditions

The normal gastricepithelial cell line, GES1 and gastric cancer cell lines including AGS, MKN45, MGC803, SGC7901 and HGC27 cell lines were obtained from the Committee of Type Culture Collection of the Chinese Academy of Sciences (Shanghai, China). All the cell lines were cultured in RPMI 1640 supplemented with 10% heat-inactivated FBS (fetal bovine serum) and 1% penicillin-streptomycin. All cells were incubated at 37°C in a humidified chamber containing 5% CO2.

### Extraction of total RNA and real-time quantitative PCR

Total RNA was extracted using TRIzol (Invitrogen, Carlsbad, California, USA) according to the manufacturer's protocol. The total RNA concentration was assessed by measuring absorbance at 260 nm using a NANO DROP spectrophotometer (ND-1000, Thermo Scientific, USA). Reverse transcription (RT) to synthesize the first-strand of cDNA was performed with 2 µg of total RNA treated with M-MLV reverse transcriptase (Promega, USA) according to the manufacturer's recommendations. The resulting cDNA was then subjected to real-time quantitative PCR for evaluation of the relative mRNA levels of TCEAL7 and GAPDH (glyceraldehyde-3-phosphate dehydrogenase, as an internal control) with the following primers: TCEAL7 forward: 5′-CGCCCCTTCCTTTTTGTTT-3′, and reverse: 5′-GGCTTTCCTTCGTTTTCTTTG-3′; TCEAL1 forward: 5′-CACTCTCCCGAAAAGCAGTC-3′, and reverse: 5′- CTGCCATGTATGTCCCCTCT-3′; TCEAL3 forward: 5′-TGGCCTTCCATTCTGATTTC-3′, and reverse: 5′-AAAGGCTGCTGGTAACGAGA-3′; TCEAL4 forward: 5′-GAAAAGGAGGGGAAATCTCG-3′, and reverse: 5′-GGCTTTCTCTCGTCTTGTGG-3′; TCEAL5 forward: 5′-TGTGCAGAAGTCCCTCTCCT-3′, and reverse: 5′-CTTCTTTGCCTTTCCTGCAC-3′; TCEAL8 forward: 5′-GTTAGGCATTTGGACCCTGA-3′, and reverse: 5′-TGCGTGAATGTCTTTGCTTC-3′; GAPDH forward: 5′-CTCCTCCTGTTCGACAGTCAGC-3′, and reverse: 5′-CCCAATACGACCAAATCCGTT-3′. Gene-specific amplification was performed using an ABI 7900HT real-time PCR system (Life Technologies, Carlsbad, California, USA) with a 15-µl PCR mix containing 0.5 µl of cDNA, 7.5 µl of 2× SYBR Green master mix (Invitrogen, Carlsbad, California, USA), and 200 nM of the appropriate oligonucleotide primers. The mix was preheated at 95°C (10 min) and then amplified at 95°C (30 sec) and 60°C (1 min) for 45 cycles. The resolution curve was measured at 95°C for 15 sec, 60°C for 15 sec and 95°C for 15 sec. The Ct (threshold cycle) value of each sample was calculated from the threshold cycles with the instrument's software (SDS 2.3), and the relative expression of TCEAL7 mRNA was normalized to the GAPDH value. The data were analyzed using the comparative threshold cycle (2^−ΔCt^) method as the following formula: Relative expression level = 2^−ΔCt^ = 2^−Ct (GAPDH)^−2^−Ct (TCEAL7)^, in which Ct(GAPDH) means the Ct value of GAPDH and Ct(TCEAL7) means the Ct value of TCEAL7. The relative expression changed more than 40% was considered as different.

### Western blotting analysis

The homogenized gastric cancer samples, including tumor and nontumor tissues, were lysed in RIPA lysis buffer, and the lysates were harvested by centrifugation (12,000 rpm) at 4°C for 30 min. Approximately 20 µg protein samples were then separated by electrophoresis in a 12% sodium dodecyl sulfate polyacrylamide gel and transferred onto a polyvinylidene fluoride membranes. After blocking the non-specific binding sites for 60 min with 5% non-fat milk, the membranes were incubated overnight at 4°C with a rabbit monoclonal antibody against TCEAL7 (PTG Company, USA, at a 1∶200 dilution). The membranes were then washed three times with TBST (tris-buffered saline with tween-20) for 10 min and probed with the horseradish peroxidase (HRP)-conjugated goat anti-rabbit IgG antibody (Immunology Consultants Laboratory, USA, at a 1∶2000 dilution) at 37°C for 1 hour. After three washes, the membranes were developed by an enhanced chemiluminescence system (Cell Signaling Technology, Danvers, Massachusetts, USA). The band intensity was measured by densitometry using the Quantity One software (Bio-Rad Laboratories, Inc. Hercules, CA, USA) and the amount of TCEAL7 protein present was further measured by densitometry and height. The protein levels were normalized to that of GAPDH detected using a mouse anti-human GAPDH monoclonal antibody (Shanghai Kangchen, China, at a 1∶10000 dilution) and a change of 20% was considered as different.

### Immunohistochemistry analysis

The tissue sections were deparaffinized with dimethylbenzene and rehydrated through 100%, 95%, 90%, 80% and 70% ethanol. After three washes in PBS (phosphate-buffered saline), the slides were boiled in antigen retrieval buffer containing 0.01 M sodium citrate-hydrochloric acid (pH = 6.0) for 15 min in a microwave oven. After rinsing with PBS, the tissue sections were incubated with the primary antibody and the slides were then rinsed in 3% peroxidase quenching solution (Invitrogen) to block endogenous peroxidase. The sections were then incubated with a rabbit monoclonal antibody against TCEAL7 (PTG Company, USA, at a 1∶100 dilution) at 4°C overnight and then incubated with horseradish peroxidase (HRP) (ChemMateTM DAKO EnVisionTM Detection Kit) at room temperature for 30 min. After washing in PBS, the visualization signal was developed with 3, 3′-diaminobenzidine (DAB) solution, and all of the slides were counterstained with hematoxylin. As negative controls, adjacent sections were processed as described above except that they were incubated overnight at 4°C in blocking solution without the primary antibody.

The specimens were analyzed by three observers (Chunyu Huang, Lin Lv, and Shunmei Yan) who were blinded to the patients' clinical outcomes. There were about 10% cases in which the three observers couldn't reach the same results at first. But after further review, they reached a consensus. The observers had a good consistency for Immunohistochemistry analysis (kappa = 0.80, p<0.001). The total TCEAL7 immunostaining score was calculated as the sum of the percent positivity (the percentage of the positively stained tumor cells) and the staining intensity. The percent positivity was scored as “0” (<5%, negative), “1” (5–25%, sporadic), “2” (25–50%, focal), or “3” (>50%, diffuse). The staining intensity (localized to the nucleus) was scored as “0” (no staining), “1” (weakly stained), “2” (moderately stained), or “3” (strongly stained). The total TCEAL7 immunostaining score ranged from 0 to 9. In previous studies, the patients were divided into 2 groups (low vs. high) based on their immunostaining score [16], [17], [18], [19]. According to these methods, the gastric cancer patients were also divided into 2 groups (low vs. high) based on the TCEAL7 immunostaining score in our study. We defined TCEAL7 expression as ‘−’ (score 0–1), ‘+’ (score 2–3), ‘++’ (score 4–6) and ‘+++’ (score >6). Thus, patients whose TCEAL7 expression levels were ‘−’ (score 0–1) or ‘+’ (score 2–3) were combined in the low expression group, and those whose TCEAL7 expression levels were‘++’ (score 4–6) or ‘+++’ (score >6) were combined in the high expression group.

### Follow-Up

The postoperative follow-up was conducted at our outpatient department and included clinical and laboratory examinations every 3 months for the first 2 years, every 6 months during the third to fifth years, and annually for an additional 5 years or until patient death, whichever occurred first. Overall survival, which was defined as the time from the operation to the patient's death or the last follow-up, was used as a measure of prognosis [20], [21]. There were about 4% patients lost follow-up.

### Statistical analysis

A paired-samples t-test was used to compare the TCEAL7 mRNA levels in the tumor tissue samples and the adjacent non-tumor tissue samples. When the variables are quantitative, we used Pearson's correlation coefficients to analyze the relationship between TCEAL7 expression and various clinicopathological characteristics. But when the variables are categorical, we use association coefficients for contingency table to analyze the relationship. Overall survival curves were calculated with the Kaplan-Meier method and were analyzed with the log-rank test. Cox proportional-hazard analysis was used for univariate and multivariate analysis to explore the effect of clinicopathological variables and TCEAL7 expression on survival. Only the factors which were found to have statistically significant associations with overall survival based on a univariate analysis would be included in a multivariate Cox proportional hazards model to adjust for the effects of the covariates. Furthermore, variables that were highly associated with others were excluded from the final multivariate Cox proportional hazards model. Proportional hazards assumption was tested by adding a time-dependent version of all variables in the model. For categorization purposes, age was entered into the model in two different groups, where “≥60” group was compared to “<60” group. Similarily, the other variables were treated as above (Table 2). A two-sided P-value<0.05 was considered to be statistically significant. All statistical analyses were performed with SPSS software (version 17.0; SPSS Inc., Chicago, IL, USA).
